# P07 Pityriasis amiantacea: a unique presentation of psoriasis associated with tumour necrosis factor-α inhibitor therapy

**DOI:** 10.1093/rap/rkad070.028

**Published:** 2023-09-27

**Authors:** Shivani Gor, Khin Yein, Elizabeth Price

**Affiliations:** Royal National Hospital for Rheumatic Diseases, Bath, United Kingdom; Great Western Hospital, Swindon, United Kingdom; Great Western Hospital, Swindon, United Kingdom

## Abstract

**Introduction:**

Pityriasis amiantacea (PA) is a rare but distinctive scalp condition. It is characterised by thick, silvery scales that bind together causing matting of multiple hair shafts. This can ultimately result in temporary or permanent hair loss. PA is thought to be a reaction pattern rather than a specific diagnosis and common associated conditions include: scalp psoriasis, seborrhoeic dermatitis, tinea capitis and atopic dermatitis. We report a case of PA presenting in a patient with longstanding rheumatoid arthritis (RA), associated with tumour necrosis factor-α inhibitor (anti-TNF-α) therapy.

**Case description:**

A 42-year-old was reviewed at her routine rheumatology follow up appointment. She has a 15-year history of seropositive, erosive RA managed with monthly golimumab injections for the last eight years. Prior to this she had been on etanercept for four years, but this lost efficacy. Unfortunately, she had poor disease control on methotrexate, leflunomide and sulphasalazine.

She developed a sore area on her scalp nine months previously with accompanying itch. This then spread into an extensive confluent area of redness, associated with soreness, scale, matting onto hair and hair loss. She had been treated unsuccessfully with multiple antibiotic courses, including flucloxacillin, doxycycline and clarithromycin, oral fexofenadine, topical diprosalic and ketoconazole shampoo. The pruritic skin lesions continued to progress and even spread to behind the ears. This had been managed by her general practitioner (GP) as otitis externa. The patient was otherwise well with no joint pains, fever, weight loss or other systemic features.

On examination, thickened, hyperkeratotic scaling plaques were seen with clumped hair shafts and underlying erythema of the entire scalp. There were no skin lesions over the rest of the body and nail changes were absent. Swab culture from the scalp was negative for fungal infection but *Staphylococcus aureus* and *Psuedomonas aeruginosa* were identified. Dermatology consult was sought, and she was diagnosed with extensive impetiginized pityriasis amiantacea associated with otitis externa. This was thought to be secondary to her anti-TNF-α therapy. Initial management was with scalp oils to soften the scales, Betnovate scalp ointment and a course of antibiotics. However, the mainstay of treatment was stopping the golimumab. Within one month of these interventions she made a dramatic improvement.

**Discussion:**

The pathogenesis of PA is yet to be definitively established; however, it has been thought that PA may be a manifestation of an underlying inflammatory skin disease. Most commonly, PA is associated with psoriasis and seborrheic dermatitis. Drug induced cases have also been described. There are case reports of PA occurring in patients treated with valproic acid, vemurafenib and minoxidil. The skin symptoms seen in PA are likely a reaction of hair-bearing scalp to various inflammatory and microbial insults. Moreover, a study undertaken in Egypt found that over 96% of patients with PA had *Staphylococcus* isolates found in skin swabs taken from the affected scalp. Both *S. aureus* and *S.epidermidis* were identified; this could suggest incidental colonisation or that these bacteria play a part in development of PA. Multiple studies have looked at distinguishing microbial biofilms and their structure and physical properties. Resilient biological scaffolding of microbial biofilms, rich in polysaccharides, proteins, lipids, and nucleic acids, is triggered by the bacteria and develops on the scalp and hair shafts. As this continues it promotes accumulation of adherent scale and the hair shafts are increasingly bound together and matted to the scalp.

TNF-a is an inflammatory cytokine produced by macrophages during acute inflammation. It causes a range of signalling events within cells, leading to necrosis or apoptosis, which contributes to the pathogenesis of many chronic inflammatory diseases, like psoriasis. However, in some cases TNF-a inhibition can paradoxically lead to new psoriasis or worsening of existing psoriasis. This has been observed with the three major anti-TNF-a agents (infliximab, etanercept and adalimumab). Often it is seen in patients receiving treatment for rheumatoid arthritis or inflammatory bowel disease. On average, cutaneous adverse effects are seen approximately 17 months after starting anti-TNF-a therapy. The most frequently seen presentations are of plaque psoriasis and palmoplantar pustulosis.

**Key learning points:**

Inhibition of TNF-a causes increased production of interferon-a by dendritic cells, which has been associated with induction of psoriasis. Interleukin-1 (IL-1) has also been implicated in the development of psoriasis. Furthermore, TNF-a can activate apoptosis of potentially autoreactive T-cells. Blockade of this action could lead to T-cell mediated tissue and psoriatic pathology.

Anti-TNF-a induced PA is a rare adverse reaction that usually requires drug discontinuation. The exact pathogenetic mechanism is unclear but an imbalance of the cytokine environment has been theorised. Our patient highlights the need to stay vigilant for cutaneous adverse reactions and the evolving diverse spectrum of psoriasiform conditions that can be seen secondary to anti-TNF-a therapy.

